# A Case of Intussusception in Which Laparotomy Was Performed

**Published:** 1878-02

**Authors:** A. A. Hubbell

**Affiliations:** Leon, N. Y.


BUFFALO

Medical and Surgical Journal.

VOL. XVII. FEBRUARY, 1878. No. 7

### Original Communications.

:o:

ART. I.—*A Case of Intussusception in which Laparotomy was performed.* By A. A. Hubbell, M. D., Leon, N. Y.

The operation of abdominal section, or laparotomy, for intussusception is rare in the annals of surgery. Its history and results were little known prior to 1874, when Prof. John Ashurst, Jr., M. D., of Philadelphia, published his valuable paper* containing the abstracts of all the cases he could then find,—only fourteen in number. Dr. Ashurst recently informed me that, at the present time, he has collected but twenty-seven cases, including those he previously published. I have references† for twenty-five cases besides my own. In the United States I can find only three cases previous to mine, viz: 1, that of Dr. John R. Wilson, reported in the *Transylvania Medical Journal*, 1835, (see also, *American Journal of the Medical Sciences*, 1836 and 1874); 2, that of Dr. John L. Ancrum, reported in the *Charleston (S. C.,) Medical Journal and Review*, October, 1874; 3, that of Dr. H. B. Sands, reported in the *N. Y. Medical Journal*, June, 1877. The remaining twenty-two cases are European and British,—mostly the latter.

---

* American Journal of Medical Sciences. † See Table appended to this Article.

A few years ago, the profession almost universally condemned the operation ; yet the basis for such condemnation must have been, in the main, hypothetical and traditional. Had medical men become acquainted with the facts and results of it, few as they were, it would not, I apprehend, have been denounced in such unmeasured terms. But the sentiment is changing, and the operation is now looked upon with more favor. The data, however, is yet insufficient to guide the surgeon in its application to intussusceptions although they may faintly mark out the general indicatious. It is with the view of adding to these data that I present the details of my own case.

Stanley H———, aged 3½ years, naturally robust and strong, was convalescent from measles in a mild form when, on the 14th of April, 1877, after sleeping well the night previous, and while playing about the house, he was suddenly seized at about 7 o'clock, A. M., with severe pain in the abdomen. He manifested great suffering which his attendants could not relieve, and I was sent for at about 9 A. M. But living some three miles from the patient, and also being absent on professional calls, I did not see him until noon. I found him very restless, throwing himself in various positions from one side of the bed to the other. At this time he showed no other signs of distress or pain, there being no moans or cries. The pulse was frequent, quick and feeble, and there was a general coldness of the surface of the body which the friends told me had been greater an hour or two previous to my arrival. His bowels had been regular and had moved naturally the day before, but not since. He had vomited several times during the forenoon after taking food or drink. A careful examination of the abdomen showed nothing abnormal. Regarding the case with some suspicion and doubt, I left a simple laxative of rhubarb with carminatives, with directions to report to me the condition of the patient in the evening. At 8 P. M., I received information that the child was about the same. No severe pain had been shown, but he continued very restless. All the medicine, drink, etc., had been vomited, and there had been no movement of the bowels. I sent a mixture consisting of ℞ Sul. of Morphia one-fourth grain, Fl. Ex. Aconite Root three drops, Water one ounce. Of this I ordered one-half to one teaspoonful to be given every one to four hours according to amount of restlessness and vomiting.

April 15th, 9 A. M. I found the patient more comfortable. He had not vomited since the anodyne was given the evening before, although only two or three doses had been administered during the night. He had shown no signs of pain, but had been quiet and apparently easy all night and had slept well. The apparent temperature of the body was natural but the pulse was somewhat accelerated. On examining the abdomen, I detected a tumor in the right iliac region. It seemed to consist of two portions, a distinct and an indistinct. The distinct portion was about three to four inches in its long diameter and an inch and a quarter transversely, and lay parallel with the axis of the child's body. The indistinct portion appeared to proceed from the upper part or end of the distinct, and was reflected to the left toward the umbilicus and then downwards. As nearly as could be felt, it, also, seemed about three or four inches in length ; yet its outlines and resistance were not sufficiently well marked to make it positive that it was a part of the tumor. The distinct portion was plain in its outlines, and abruptly terminated at the beginning of the soft, indistinct portion. It was hard and resisting, dull on percussion, somewhat tender on pressure, and more or less movable. There was no general tenderness of the bowels, no tympanitis, no disposition to flex the thighs, and any position of the body was assumed without uneasiness or distress. There had been no movement of the bowels or desire for such. Whether I had a case of obstruction from invagination or other cause, or one of typhlitis, of which I had recently had three well marked cases with similar symptoms, except the violence of the attack, I could not decide. I therefore concluded to continue the anodyne solution and await further symptoms.

5 P. M. There had been more vomiting, and occasional signs of pain in the afternoon. Otherwise the symptoms were about the same. I ordered the anodyne in larger and more frequent doses, heretofore, only one-half teaspoonful every three to four hours having been necessary.

On the morning of the 16th I was informed that the patient had beeu somewhat restless through the night, and had given occasional evidence of a little pain. There had been some thirst, but no appetite, and he had vomited several times after taking drink. The bowels remain constipated, and in every way nearly the same as on the day previous. At this time I thought I would try an injection, and see if I could get a passage from the bowels. I used warm water stimulated with soap. The result of the injection was that it was returned emitting a very fœtid odor, and considerably colored with blood, and containing a trifling quantity of hardened feces and a little bloody mucus. The character of this evacuation, together with the symptoms previously noted, seemed to me a sufficient basis for now determining a diagnosis of intussusception. The location of the tumor led me to believe that it was the variety that authors term ileo colic, in which the ileum passes into the colon through the ileo-cœcal valve, or ileo-cœcal in which the cæcum is inverted into the colon carrying with it the ileum, although I feared that it was within the small intestine. I hoped that the constriction might not be great, that the impaction and incarceration might not be severe, from the fact that, since the first onset of the symptoms, they had assumed a mildness that I could not expect with tightly involved structures, and that could not be accounted for by the use of the anodyne mixture, as it had been given in too small doses to overcome severe pains, vomiting, etc. I therefore resorted to that most potent and hopeful of all remedies for intussusception,—distention of the colon—with a good deal of cheer and faith that some good result might accrue. I first tried inflation with air, using the only instrument immediately at my disposal, a Matteson's family syringe "No. 1." to which I attached the vaginal tube. I introduced the tube the full length into the rectum, around which with one hand I compressed the nates, while with the other I worked the syringe in the usual manner, and gradually and thoroughly distended the colon with air to the fullest extent that the patient would bear. The distention was continued for a short time, while the abdomen was gently kneaded over, and around the tumor. The air was then allowed to pass off, but no change in the tumor was perceptible. After a short interval the inflation was repeated, but with the same eff ect

—no change. After the inflations the patient desired to use the stool, and two or three times passed one to two fluid drachms of bloody mucus,—additional confirmation of invagination.

Next, I tried full injections of warm water, but without any effect upon the tumor. The repetition and alternation of different modes of distention failed to accomplish any good, and I felt that this was to be a hopeless mode of procedure. There was but one thing else that presented itself to my judgment as holding forth any prospect of saving the patient's life, and that was the operation of abdominal section. Although the operation was one of, perhaps, a dangerous character, and one very generally condemned by the profession, yet, in case reduction could be accomplished there would be some chance of recovery; if it could not be accomplished death would take place, which would only be the same result as that which would follow were the case left to nature;—death was very certain unless reduction could be effected by means of art. Having failed, then, in accomplishing anything desirable by a reasonable use and repetition of insufflations and injections, I deemed it expedient, and a duty to give the child the only chance that I could see for his life, meager though it might be, and operate as early as possible by abdominal section. Before venturing such a procedure I desired advice in the matter, and another physician was called. He arrived at about 11 o'clock, P. M. On examination of the case he agreed with me in diagnosis. He advised another trial of distention of the colon, and it was used several times without success, the child being partially under the influence of chloroform. I then represented to him my opinion of the means that should be resorted to and urged the propriety of an immediate operation, as all other measures had failed, and in my judgment would continue to fail. And as to hoping for recovery at his age by sloughing of the incarcerated intestine, or otherwise, I considered it most vain. But he did not agree with me. He regarded the operation as a hazardous and fatal procedure, and emphatically discouraged it. If distention did not succeed in reduction, he thought the best chance for the patient's life was to leave the case to nature. I finally yielded to the discouragement, and to the sentiment so universally prevalent in the profession, that "abdominal section for invagination is a *fool-hardy* operation." Distention of the colon was occasionally repeated to the fullest extent, the patient being under the influence of chloroform each time. The anodyne mixture was also continued.

On Tuesday the 17th, the abdomen was slightly tympanitic, but not tender on pressure except over the tumor. He vomited almost everything taken. He was restless and showed occasional symptoms of distress, but no severe pain. Strength was failing quite rapidly. I continued the use of anodynes, and also tried inflation several times without effect.

18th. Same symptoms continue, though in a more pronounced form. The tympanitis was quite considerable, the convolutions of the small intestines showing through the walls of the abdomen. His strength continued to fail. There was great restlessness, but not any severe pain. Vomiting became stercoraceous about 10 A. M., and there was every indication by the feebleness of the patient, the condition of the circulation, etc., that death was approaching with rapid pace. I was certain that the child must die as he was, and that even at the lateness of the hour of the disease, if reduction could possibly be accomplished by an operation, recovery might take place; at least, this was the *only* hope, and having the consent of the friends I would avail myself of it without further delay. I had some hope of accomplishing reduction, for the reason that, to my mind, the symptoms had been those of simple obstruction without severe constriction or strangulation, or indications of inflammation and adhesions. Dr. F. C. Beals, of Conewango, N. Y., was called to assist me in the operation.

The patient being well under the influence of chloroform, I proceeded with the operation at 7 P. M.,—four and one-half days after the first manifestation of symptoms. To facilitate an exploration to determine the correctness of our diagnosis, I operated directly over the tumor, making an incision parallel with the right rectus muscle, and about midway between its outer border, and the anterior superior spinous process of the right ilium, and of sufficient length to admit a finger. On passing my index finger into the cavity of the abdomen I at once came upon the tumor, a careful examination of which proved the correctness of the diagnosis of invagination. Removing my finger, I enlarged the opening to three or four inches in extent, the inferior extremity being a little lower than the anterior superior spinous process. Through this opening I readily brought outside the intestines in which the invagination was situated. As I had previously feared, it was wholly within the small intestines, the lower part of the ilium being the portion involved. The tumor measured about seven inches in length and one and a half inches transversely. The distance from the ileo-cæcal junction to the "mouth" of the invagination was nearly twelve inches. The mesentery was tightly drawn in with the entering portion of the bowel, and both were firmly impacted within their sheath. The mouth of the tumor was much constricted, the enclosed bowel being reduced to a tendinous cord about the size of a goose-quill. The bowel and mesentery within the mouth for three or four inches in extent were swollen and hard, while the remaining three or four inches were soft and loose (movable) within the sheath and did not appear much if any swollen. The outside or ensheathing portion of the intussusception was swelled, deeply congested, and ecchymotic, and the peritoneum and tissues beneath seemed softened. The ileum for five or six inches above the point of invagination, and the mesentery adjoining and for some distance around it were also much congested. There were no adhesions outside of the invagination, and from the fact that the portion within was movable for half of its extent, and that, with care, I could pass my finger for some distance into the mouth, and entirely around the entering bowel, I inferred that there were none within. But considering the degree of incarceration, induration and infiltration of the bowel and mesentery within, as well as of the bowel without, together with the severe constriction of the hardened and swelled mouth, and the softened condition of the tissues around, the prospect of reduction looked dark, indeed. The attempt, however, was made by Dr. Beals grasping the ileum above the invagination, and gently making traction upon it while I manipulated the tumor from below by carefully squeezing and compressing the enclosed portion upwards and drawing the sheath downwards, thus endeavoring to turn the mouth outwards, and "back out" the bowel and mesentery from within. We continued and varied our manipulations for some time, and with as much force as we dare use, without any success. The softened peritoneum over the tumor tore during these maneuvers. While making another attempt with more force, though not great, a rent about a quarter of an inch in length took place in the softened bowel just above the tendinous, constricted portion, with some extravasation of the contents of the intestines. We continued our efforts a while after this, but availing nothing finally desisted. We did not deem an artificial anus or excision of the invagination of any use, and, therefore, after ligating the torn bowel, and returning the intestines into the cavity of the abdomen with some difficulty, we closed the wound with sutures and adhesive plaster, and gave up the case to die. A little less than an hour was used from the beginning to the close of the operation, including the giving of the chloroform. As the patient rallied to consciousness he manifested severe pain, which was somewhat releived by a hypodermic injection of Sulphate of Morphia. At about 11 P. M., having, for an hour or two previous, shown signs of much distress and dyspnœa between intervals of sleep, he was seized with a convulsion. Although it was not severe or long, he relaxed in death. Thus ended the suffering of our little patient.

No Post-Mortem examination was made.

*Remarks*—This case illustrates that there may be severe constriction, much swelling, firm impaction and great strangulation without the symptoms being correspondingly exaggerated, there having been but little tenderness, and the pain after the first three or four hours having been so slight as to be controlled by minute doses of morphia, and the vomiting not having been at all urgent till the third or fourth day. The obstruction was complete, as might have been expected, had the invagination been known to be in the small intestine; there was no tenesmus. It is true, therefore, that the condition of the invagination cannot be determined by the symptoms.

This case, also, illustrates that a simple injection may determine a doubtful diagnosis by bringing away blood and bloody mucus, which indicates so characteristically intussusception. Further, there may be great congestion, swelling, infiltration and even softening without adhesions taking place at the expiration of four and one-half days. Reduction may also be impossible by simple manipulation, though the length of the invagination be not great, and there be no adhesions.

The question naturally arises, could reduction have been accomplished at the time when the diagnosis was first made,—forty-eight hours after the attack? The infiltration, swelling and softening must have been less, and consequently there was less firm impaction; the tissues must have been stronger, and consequently more force could have been used; thus the chances of reduction must have been greater, and I believe it might have been accomplished.

Again, could any other means have been used to effect reduction?

There is one other measure that would commend itself to my judgment, and which I should use were the case to be repeated. Having failed in reasonable manipulation, and stopping short of producing any abrasions or rents of tissues, I would relieve all constriction by making an incision through the sheath of the invagination, beginning at its mouth and extending longitudinally sufficiently far to admit of the indrawn mesentery and bowel being extricated and returned to their natural relationship, the wound in the intestine being afterwards closed by sutures. Excision of the invaginated bowel has been resorted to (see cases No. 22 and 24 in appended table) and the ends of the divided healthy intestine stitched together, or to the edges of the abdominal wound. If there were no mesentery this would seem a feasible operation, as a last resort. But there must be great danger of hemorrhage from wounding, as would be necessary, such a vascular structure, to say nothing of other sequelæ that might attend it. And though the procedure might not be wholly unjustifiable in certain cases, yet I do not feel that there is much to commend it. The establishment of an artificial anus is another operation that might be doubtfully considered, but without any facts to encourage its use. Supplemented, therefore, to simple laparotomy, and after unsuccessful manipulation, I consider cutting through the sheath from its mouth, the most hopeful procedure when there is no gangrene or destruction of tissues, and would resort to it were the conditions of the above case to be repeated.

In conclusion, I wish to express my opinion founded upon a careful study of the subject that, as a rule, in acute cases of intussusception, when other remedial measures have failed, after a thorough and reasonable trial, laparotomy ought to be performed at the earliest possible moment. An early diagnosis is an important element in a successful result, as well as an early operation. In chronic cases an operation resorted to as soon as other means are of no avail will give the patient the best chance for life. Indeed, in all cases, where all other resources reasonably tried have been unsuccessful, and especially where there is obstruction, I believe an *early* use of the operation will greatly enlarge the present percentage of recoveries. And even when there is not complete obstruction, it is so liable to take place at any time, and adhesions are so apt to occur, that, when a diagnosis is positive, and reduction by other means is impossible, I deem laparotomy a justifiable and proper procedure. In resorting to it, however, I would use careful discrimination, and except those cases in which sloughing, or other serious organic changes were taking place in the involved structures.

The most general survey of statistics* substantiates my opinion, and holds out great encouragement for the use of the operation. According to Leichtenstern, the mortality of all cases taken together is 73 per cent. Of infants, his statistics show a mortality of 86 per cent. whether subjected to treatment or not. Dr. J. Lewis Smith's analysis of 50 cases gives a mortality of 86 per cent.

Dr. Ashurst, Jr., in a private letter to me, states that he has collected 27 cases in which laparotomy has been performed. Of these 9 recovered, 17 died, and in one the result was not known. I have references for 26 cases, including Dr. Ashurst's cases of 1874, 8 additional cases referred to by Dr. Sands in 1877, *Carrier's case mentioned by Hutchinson* in 1874, Dr. Ancrum's case reported in

* For a more minute analysis of statistics, see Dr. H. B. Sands' paper in New York Medical Journal, June 1877.

October 1774, Dr. Howard Marsh's case published in Vol. XII of St. Bartholomew's Hospital Reports, and my own case hereby reported. Of these 26 cases the result was known in 24, of which 8 recovered and 16 died, thus giving a mortality of $66\frac{2}{3}$ per cent.—the same as that of Dr. Ashurst's cases with my case included.

This general analysis of statistics indicates an apparent gain in recoveries of 6 to 20 per cent. Indeed, comparing the non-operative with the operative treatment, the latter saves more lives by from 6 to 20 per cent. than the former. Looked at, however, properly, the operative measures save a certain number that must die with other treatment, and therefore the *real gain is in all those saved by* laparotomy. Is it "fool-hardy," then, to operate? Indeed, such a sentiment is unfounded! And when the time comes when it is used in all reasonable cases, after other means have failed, and where an early diagnosis has been made, statistics will show a greatly increased percentage of recoveries over those of today. Contrary to the opinion of Dr. Ashurst expressed in 1874, I believe, also, that there is much encouragement for operating on infants, from the fact that without an operation 86 per cent. die, while with it, 82 per cent. of those subjected to it die, making the latter the safest treatment by at least 4 per cent. According to Dr. J. Lewis Smith and others, all cases in infants, that cannot be remedied (reduced) by non-operative measures, must die, if left to nature. All cases operated upon are considered irremedial by all other treatment. Therefore, all those that recover by laparotomy are so much clear gain of life; and the percentage of recoveries from intussusception is to that extent increased. Without laparotomy, then, only 14 per cent. recover, while with it, by saving 18 per cent. of the remaining 86 that must otherwise have died, there is the pleasing proportion of 30 per cent. who recover.

A fair comparison of statistics, therefore, leads to the necessary conclusion that laparotomy is a potent means of saving life in intussusception, and should not be delayed after other remedies prove unavailing; and especially should it be used in *infants* for after other measures fail, this is *their only chance* for life. Mr. Howard Marsh* well expresses my opinion when he says:—"If the

* St. Bartholomew's Hosp. Rep. Vol. XII.

diagnosis is complete, if strangulation is recent, say of not more than eighteen hour's duration, or if, in chronic cases, there is room for good hope that inflammation is not present; if other means of reduction have failed, and if there is no other circumstance in the case that makes failure a foregone conclusion, or leaves little less hope of success, the operation should be performed, after the common rule in hernia, at once. Till this becomes the accepted practice in these cases, it will never be known how many lives among them may be saved by abdominal section." And with Dr. Ancrum I would add:—"Where, and whenever death, without interference, seems inevitable, not only is the surgeon justifiable, but he is emphatically culpable if he shrinks from the responsibility of any operation which offers to a fellow being the remotest chance of life."

The following table shows the statistics of the subject so far as I can reach them :

### TABLE OF CASES.

| No. | Operator. | Sex. | Age. | Symptoms. | Duration before Operation. | Location. | Condition of Parts at Operation. | Reduction. | Result. | Reference to Case. |
|---|---|---|---|---|---|---|---|---|---|---|
| 1 | Mil Surgeon, | Female, | | Those of "Ileus." | | | | App'ly easy | Recovery | Bonetus Sepulch. t. ii. p. 228, 1700. |
| 2 | Surg. by Nuck | " | 50 years | "Worn out with the symptoms of this most cruel disease," (intussusception.) | | | | " " | " | Haller, Dusputat, Anat. Sel., 1751. |
| 3 | Ohle | Male, | 50 years | Protrusion of intestine beyond anus, pain, vomiting, tenesmus, hemor. from bowels. | 11 days | L. intestine | Adhesions. | Difficult | Death in 12 hours | J. N. Rust's Magazine, 1817. |
| 4 | Fuchsius, | " | 28 years | Tumor near umbilicus, pain, vomiting, constipation, | 10 days | | No Adhesions | Easy | Recovery | Hufeland and Osann's Jour. 1825. |
| 5 | Wilson, | " | 20 years | Stertoraceous vomiting, intestinal obstruction, | 17 days | Ileum | Quite firm Adhesions | Difficult | " | Transylvania Jour. of Med. 1835. |
| 6 | Gerson, | " | 12 weeks | Tumor in left inguinal region and rectum, intestinal obstruction and hemorrhage | | L. intestine | Firm Adhesions and Gangrene | Impossible. | Death in few hours. | Fricke and Oppenheim, 1840. |
| 7 | Hauf, | " | 36 years | Tumor in abdomen and outside anus, pain, intestinal obstruction and hemorrhage, | 4 or 5 days | " | Peritonitis & Adhes'ns | " | Death in 9 days | Heidelberg Med. Annal. 1843. |
| 8 | Pirogoff, | " | 16 years | Symptoms of obstruction, | Consid'able | | Bowel Gangrenous | Not attemp. | Death shortly | St. Petersburg, 1852. |
| 9 | Wells, | | 4 months | Tumor felt in rectum, symptoms of intussusception, | 4 days | L. intestine | No Adhesions, child almost Moribund | Effec'd with difficulty | Death in 5 hours | Trans. Path. Soc. of London, 1863. |
| 10 | Laroyenne, | | | | | | Adhesions | Impossible. | Death — — | Servier De l' Occlus. Intestinale,'70 |
| 11 | A. Johnstone, | | Child | | | | | | Death — — | Lancet, (London,) Feb. 11, 1873. |
| 12 | Weinlechner, | Female, | 6 months | Tumor in left hypogastrium, pain, vomiting, passage of blood and bloody mucus, | 3 days | L. intestine | | Very diffic't | Death in 6 hours | Schmidt's Jahrbucher, 1872. |
| 13 | Hutchinson, | | 2 years | Symptoms of obstruction, protrusion of bowel from anus, | 1 month | " | No Adhesions, | Easy | Recovery | Med. Chir. Trans. Vol. lvii. 1874. |
| 14 | Carrier, | | | | | | | | | Hutchinson, in Med. Chir. Transactions. Vol. lvii. 1874. |
| 15 | Duncan, | | 5 months | | | | | | Death in 2 days | Edinburg Med. Jour., June, 1874. |
| 16 | Ancrum, | | 11 weeks | Watery evacuations, intestinal hemorrhage, pain and tympanitis, | 4 days | L. intestine | Congestion, swelling, Oedema & Adhes'ns | Difficult | Death in 2 hours | Charleston, (S. C.) Med. Journal, Oct. 1874. |
| 17 | Marsh, | Male, | 7 months | Bowel protruded from anus, acute symptoms 12 hours before operation, | 14 days | " | No Adhesions ment'd | Easy | Recovery | Med. Chir. Transactions, 1876. |
| 18 | Howse, | Female, | 33 years | Abdominal tumor, pain and vomiting, | 18 days | " | No Adhesious | " | " | " " " " |
| 19 | Hutchinson, | | 6 months | Tumor in abdomen and rectum, pain, vomiting, tenesmus, disch. of bloody mucus, | 4 days | " | No Adhesions | Difficult. | Death in 8 hours | " " " " |
| 20 | Bell | Male, | 16 months | Tumor in abdomen and rectum, pain, vomiting, tenesmus, disch. of bloody mucus, | 5 days | " | Congested and irreducible | Impossible, made artificial Anus | Death in 7 hours | London Lancet, Jan. 1st, " |
| 21 | | | 9 months | Bowel protruded from anus, retching, discharge of bloody mucus | 7 days | " | | Easy | Death in few hours. | British Med. Jour. Sept. 16, 1876. |
| 22 | Howse, | | 5 months | Bowel protruded from anus, discharge of bloody mucus, | 1 month | " | Softened | Impos., excised 4 inch. of bowel | Death in few hours | Med. Chir. Transactions, 1876. |
| 23 | Marsh, | | 9 months | Weakness of patient, bowel protruded from anus, | 1 month | " | No Adhesions | Easy | Death in 10 hours | St. Bartholomew's Hosp. Rep., Vol. xii, 1876. |
| 24 | Morris, | Male, | 12 years | Tumor in rectum, pain, vomiting, constipation, passage of blood, | 7 days | " | Sloughing | Not att'pt'd 17 in. bowel rem'v'd, &c. | Not known | London Lancet, Jan. 20, 1877. |
| 25 | Sands, | Female, | 6 months | Tumor in abdomen and rectum, vomiting, pain, tenesmus discharge of bloody mucus, | 18 hours | " | No Adhesions | Easy | Recovery | N. Y. Med. Journal, June, 1877. |
| 26 | Hubbell, | Male, | 3½ years | Tumor in right iliac region, pain, vomiting, constipation, discharge of bloody mucus, | 4½ days | Ileum | Induration. Swelling, Softening | Impossible. | Death in 2 hours | Herewith Reported. |

